# Supplementary material for: Improvement of RT-DETR model for ground glass pulmonary nodule detection
Source: PLoS One. 2025 Mar 11;20(3):e0317114. doi: 10.1371/journal.pone.0317114 (PMC11896049; doi:10.1371/journal.pone.0317114)
Supplement: Attachment — Submitted filename: Response to Reviewers.docx [file pone.0317114.s001.docx]

***Response to Reviewer 1***

**1. How does the introduction of FCGE blocks improve both computational efficiency and detection accuracy for small and blurry ground glass nodules?**

**Response:**(1)FCGE module is a new module built upon deep fusion of the FasterNet module, ELA Attention module, and ConvGLU module, which has the advantages of each module.(2) PConv in the FasterNet module applies convolutional operations to only a portion of the channels of the input features, while the other channels remain unchanged, which effectively reduces the amount of computation and memory access. (3) The ELA attention module uses strip pooling in conjunction with 1D convolution and group normalization to enhance local feature representation and improve the model's detection accuracy for small-sized targets. Specifically, 1D convolution captures localized sequence signals and pinpoint target location; group normalization improves computational efficiency; and strip pooling captures long-distance spatial dependencies and captures a wider range of feature information. (4) The ConvGLU module is a hybrid mechanism that combines Gated Linear Unit (GLU) and Depthwise Convolution (DWConv). The gating mechanism of the ConvGLU module can flexibly adjust the channel response according to the blurriness of the target edges, ensuring the model focusing on the target's key features even when the target's contour is not clear. With DWConv, ConvGLU can capture detailed features in localized areas, including edge information, which is particularly important for identifying targets with blurry edges.(5) The ablation experiments in this paper show that the detection accuracy and computational efficiency of the model are improved by adding the FCGE module.

**2. Could you elaborate on how the HiLo-AIFI Module reduces computational redundancy while maintaining high performance?**

**Response:** (1)The HiLo-AIFI module reduces computational redundancy mainly through the HiLo module, which divides the MSA layer into two paths: high-frequency attention and low-frequency attention. HiLo splits the same number of heads into two groups proportionally by assigning $\left( 1-\alpha\right){*N}_{h}$ heads to Hi-Fi and $\alpha N_{h}$ heads to Lo-Fi, where $N_{h}$ denotes the total number of heads in the self-attention layer and $\alpha$ the split ratio. This approach allows the model focusing on features that are more critical to the task, rather than distributing computational resources evenly across all features, thus improving computational efficiency. The HiLo module assigns features to different heads, each of which focuses on the input features, which reduces redundant computation between heads. (2)The HiLo-AIFI module remains performant with the HiLo module. Hi-Fi in the HiLo Attention module focuses on the high-frequency regions of the image, containing large amount of detailed information. It enhances the expression of local features through the mechanism of local window self-attention, which can capture the details of the internal texture of the lung nodules and the homogeneity of the internal densities of the lung nodules. This helps recognizing the differences in internal density. Hi-Fi and Lo-Fi are used to extract and analyze the features of different areas, thus maintaining the high performance of the model.

**3. Can the model be adapted to detect other types of abnormalities in medical imaging, or is it specifically tailored for ground glass nodules?**

**Response:** The model is currently only applicable to the detection of ground-glass lung nodules and is still under investigation for the detection of other abnormalities in medical imaging.

***Response to Reviewers 2***

Following changes has been made to the paper in response to the feedback of reviewer 2. (1) We have added one paragraph in the introduction section that shows the potential value of deep learning models in the field of lung nodule detection. (2) Summary of innovation points of each module (FCGE, Hi-Lo-AIFI, DGAK) have been added. (3) Four additional deep learning models have been added in the performance evaluation section to compare with the improved model.
